# JAK inhibition may prevent drug hypersensitivity reactions

**DOI:** 10.1172/jci.insight.200914

**Published:** 2026-04-08

**Authors:** Xiangmei Hua, Pranali N. Shah, Gustavo A. Velasquez, Lillian Sidky, George A. Romar, Lydia W. Boer, Natalie Hickerson, Tracy Qiying Cui, Federico Repetto, Abigail Waldman, Marilyn G. Liang, J. Paul Marcoux, MacLean Sellars, Victor Barrera, Birgitta A.R. Schmidt, Arash Mostaghimi, Ruth K. Foreman, Christine G. Lian, Sherrie J. Divito

**Affiliations:** 1 Department of Dermatology and; 2 Department of Pathology, Brigham and Women’s Hospital, Boston, Massachusetts, USA.; 3 Department of Dermatology, Boston Children’s Hospital, Boston, Massachusetts, USA.; 4 Medical Oncology, Dana-Farber Cancer Institute, Boston, Massachusetts, USA.; 5 Bioinformatics Core, Department of Biostatistics, Harvard T.H. Chan School of Public Health, Boston, Massachusetts, USA.; 6 Department of Pathology, Boston Children’s Hospital, Boston, Massachusetts, USA.; 7 Department of Pathology, Massachusetts General Hospital, Boston, Massachusetts, USA.

**Keywords:** Dermatology, Immunology, Allergy

**To the Editor:** Drug hypersensitivity reactions (DHRs) are a major clinical problem with potential for significant morbidity and mortality. Presentation ranges from a pruritic rash (maculopapular exanthem [MPE]) to life-threatening reactions, including Stevens-Johnson syndrome/toxic epidermal necrolysis (SJS/TEN), characterized by skin and mucosal blistering, and drug reaction with eosinophilia and systemic symptoms (DRESS), notable for potentially severe organ disease. There are several limitations to clinical care. Whether a patient will progress to severe disease is unknown, there is no agreed upon treatment, it is often unclear to what drug a patient reacted, and there is no validated test to identify a culprit drug (1, 2). Consequently, the standard of care is typically to discontinue all possible culprit drugs and never rechallenge, even if a possible culprit drug is potentially life-saving. Recently, this standard has been challenged, either anecdotally in patients with cancer or in patients with HIV and tuberculosis when the culprit drug and/or risk of progression to severe DHR is questionable (3, 4). In the latter, stepwise rechallenge has been tried with some patients tolerating the needed drug. The rechallenged drug is promptly discontinued, and systemic steroids started upon first signs of a reaction (5). Anecdotal cases and case series suggest that systemic steroids can effectively abort a reaction with this approach (5). While a major advancement, patients are at risk for a reaction, and those that do react can no longer receive the potentially life-saving medication. An alternative approach is needed.

JAK-STAT activation has been observed in SJS/TEN, DRESS, and MPE (6–8), and JAK inhibitors (JAKi) have been successfully used in DRESS (7, 9, 10) and SJS/TEN (6). The JAKi ruxolitinib is an approved treatment for acute graft-versus-host-disease (GVHD), which can present identically to DHR. Ruxolitinib is now being trialed in GVHD prophylaxis. Furthermore, JAKi prevented SJS/TEN in mouse models, including a humanized mouse rechallenge model (6). Herein, we present three clinical cases of DHR where prophylaxis with JAKi allowed successful rechallenge to cancer therapies along with translational data supporting JAKi prophylaxis in DHR.

HLA-B*57:01 transgenic (tg) or control mice were prophylaxed with tofacitinib, ruxolitinib, or vehicle before treatment with abacavir (Supplemental Figure 1A; supplemental material available online with this article; https://doi.org/10.1172/jci.insight.200914DS1). Abacavir can induce DHR in humans expressing HLA-B*57:01 (11). Abacavir-treated HLA-B*57:01tg mice develop a drug-specific CD8+ T cell–mediated MPE-like dermatitis (12, 13). Prophylaxis with JAKi prevented development of MPE-like dermatitis, as demonstrated clinically, histologically, and by reduced ear thickness (Figure 1, A–C). Decreased DC skin infiltration and maturation (Figure 1D) and reduced skin infiltration of total immune cells, antigen-presenting cells, and T cells (Supplemental Figure 1B) were observed, including near-complete absence of skin infiltrating effector CD44hiCD62LloCD8+ T cells (Figure 1E).

In humans, differential gene expression analysis comparing individual forms of DHR to healthy skin supported JAK-STAT activation, with significantly increased transcription of JAK2, JAK3, STAT1, and STAT2 (Supplemental Figure 2A). Tissue staining for activated protein confirmed increased expression of phosphorylated JAK1, JAK2, and JAK3 and STAT1 and STAT2 in all three forms of DHR compared with that of healthy controls (Supplemental Figure 2, B–F).

Three patients with varying severity of DHR were prophylaxed with JAKi prior to rechallenge with cancer treatment thought to be causative in their DHR. All patients had aggressive tumors without alternative treatment options. Each would not have been considered for rechallenge in the absence of prophylaxis. A 64-year-old male with lung adenocarcinoma developed a progressive pruritic rash with focal burning pain and peripheral eosinophilia 5 weeks after starting lorlatinib (Figure 1F). He had no other drug exposures or signs or symptoms of infection. Dermatologic examination and histology supported MPE with features concerning for potential progression to severe DHR (Figure 1G). Systemic steroid taper did not resolve the rash. Lorlatinib was discontinued. The patient was treated with highest-potency topical steroids with resolution of rash and eosinophilia. Given the absence of available cancer treatment options, ruxolitinib 5 mg twice daily was initiated. Two weeks later the patient started lorlatinib 25 mg daily (one-quarter dose), which was increased to 50 mg and then 75 mg over 2 months. After 6 weeks without DHR recurrence, ruxolitinib was tapered to once daily. The patient experienced transient eosinophilia. After normalization of eosinophilia, ruxolitinib was tapered to once every other day, then discontinued. The patient remained on lorlatinib for another 10 months. Due to tumor resistance, dosing was increased to 100 mg daily without DHR but ultimately was discontinued given tumor progression.

A 69-year-old woman with radiation-associated angiosarcoma developed a paclitaxel-induced DHR that recurred more severely after subsequent dosing, with features that raised concern for potential progression if rechallenged (Supplemental Figure 3, A–C). Given the aggressive nature of angiosarcoma and her initial response to paclitaxel, the patient started ruxolitinib 10 mg twice daily prior to rechallenge with full-dose paclitaxel to minimize the chance of a reaction and treatment discontinuation. The patient tolerated rechallenge without signs or symptoms of DHR. The patient ultimately self-discontinued ruxolitinib, tolerating 4 chemotherapy treatments thereafter without reaction, and then underwent radical surgical excision with tumor clearance through 36 months follow-up.

IHC staining of both patients’ active DHR skin specimens confirmed JAK-STAT activation (Figure 1H and Supplemental Figure 3D).

A 54-year-old female (Supplemental Figure 4) with metastatic uterine carcinosarcoma developed a maculopapular rash 2 weeks after starting carboplatin, paclitaxel, and pembrolizumab, which resolved with topical steroids. Following cycle 2 of that regimen, the rash recurred. The patient then received carboplatin, paclitaxel, and trastuzumab and progressed to SJS/TEN. Five months later, she developed a rash consistent with early SJS following 1 dose of carboplatin with no other drug exposures or signs or symptoms of infection, indicating carboplatin culpability. Without alternative treatment options the patient initiated tofacitinib 5 mg twice daily and then tolerated 2 doses of cisplatin. Tofacitinib was held owing to upper respiratory infection. Two days later, the patient developed skin-limited DHR managed with topical steroids. Kidney disease prevented further chemotherapy, and she was transitioned to hospice care.

Though standard of care in patients with or at risk for severe DHR is to stop all possible culprit drugs and not rechallenge, this presents a major dilemma for patients without alternative treatment for their underlying disease. It is possible that none of these patients would have reacted to the rechallenged drug and that the JAKi was biologically unnecessary. However, each had sufficient evidence pointing to the presumed culprit drug and risk for severe reaction upon rechallenge such that they would not have otherwise been offered rechallenge and a chance at survival. This scenario is not unique. Patients worldwide are denied rechallenge based on a presumption of drug culpability and risk of reaction, as there is no validated test for drug culpability in delayed-type DHR.

All 3 patients were thoroughly advised of the risk of SJS/TEN and DRESS, including associated morbidity and mortality. All 3 chose to risk severe reaction rather than certain death from cancer. Moreover, all 3 oncology teams reported no contraindications from comorbidities or potential side effects, including concern for blunted tumor response to cancer treatment from the JAKi.

The enclosed data by no means definitively demonstrate mechanism, clinical efficacy, or safety, and this approach should only be considered when potential benefit clearly outweighs potential risk and in situations where patients can be closely monitored and intervened upon rapidly if necessary. Here, tofacitinib, a pan-JAKi, or ruxolitinib, a JAK1/JAK2 inhibitor with effect against JAK3 and TYK2, were used to bypass the limited immunopathologic data available regarding JAK/STAT activation in DHR. Deeper interrogation of JAK/STAT pathways in a larger number of patients across the breadth of DHR phenotypes should be performed to determine which JAKi, or other immunomodulatory agent, is best suited for this purpose. Multidisciplinary discussion and, ultimately, a prospective study testing the true potential and best approach of prophylaxis in DHR are clearly necessary but warranted. If effective, JAKi prophylaxis could completely shift care for many patients with DHR.

For detailed methods, information regarding sex as a biological variable, statistics, study approval, data availability, and acknowledgments, see the supplemental materials.

## Author contributions

Study design: SJD, XH, and PNS. Experimental planning and execution and data acquisition: SJD, XH, PNS, GAR, GAV, LS, LWB, TQC, VB, and FR. Patient/individual acting as control sample collection, processing and/ or medical record data collection: SJD, GAR, NH, AW, MGL, JPM, MS, GAV, CGL, BARS, AM, and RKF. Statistical analysis and/or mathematical modeling: VB, XH, and PNS. Manuscript preparation: SJD, XH, PNS, NH, GAV, and VB. The co–first authors contributed equally to this work. The order of co-first authorship was determined based on the relative contributions to data acquisition and manuscript preparation.

## Funding support

This work is the result of NIH funding, in whole or in part, and is subject to the NIH Public Access Policy. Through acceptance of this federal funding, the NIH has been given a right to make the work publicly available in PubMed Central.

- NIH grants DP5 OD023091, R21 AI150657, and R01 AI181221 (to SJD).

## Supplementary Material

Supplemental data

Supporting data values

## Figures and Tables

**Figure 1:**
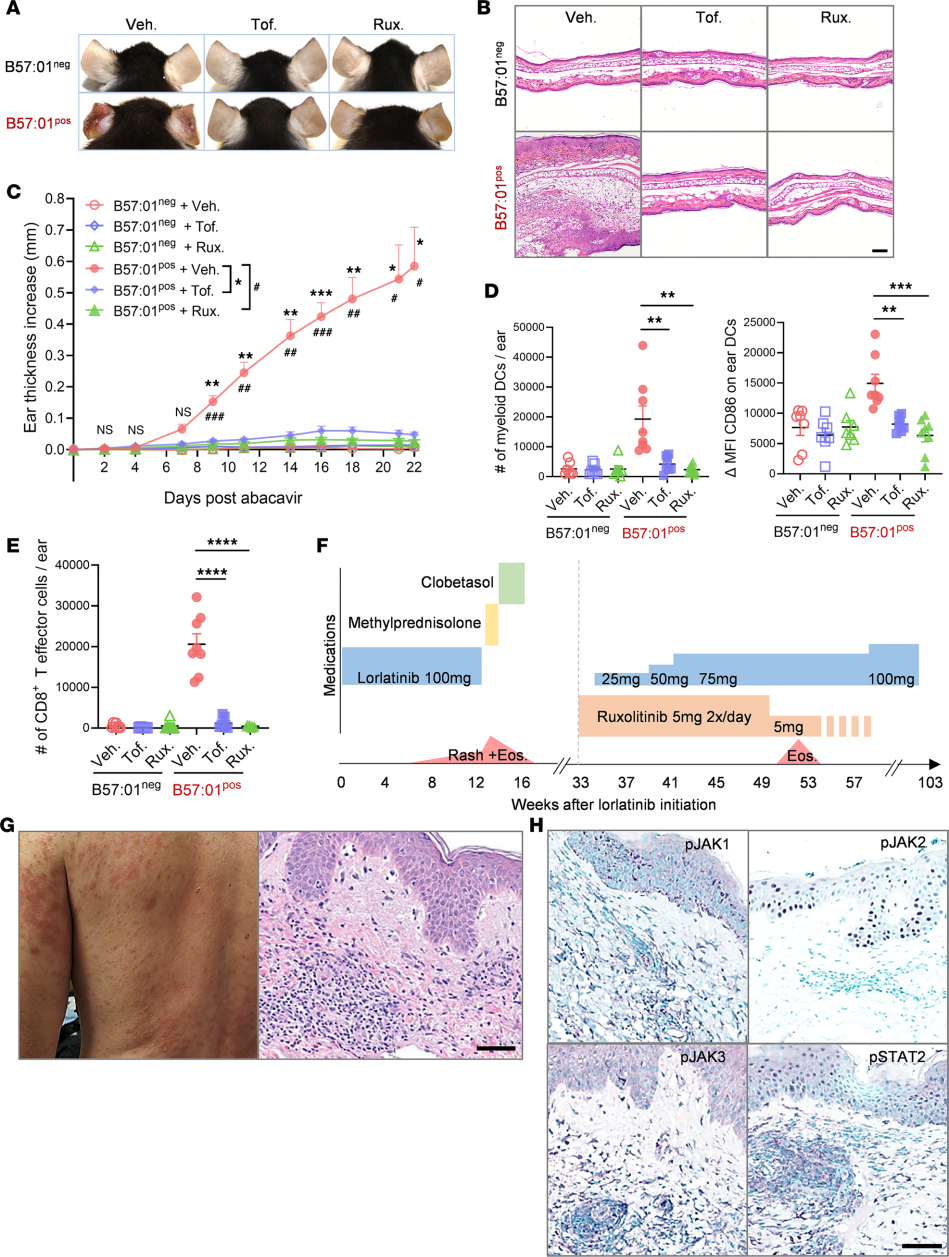
JAKi prophylaxis may prevent DHR in mice and humans. (**A** and **B**) Representative (**A**) gross and (**B**) histologic ear images of HLA-B*57:01^pos^ and HLA-B*57:01^neg^ mice prophylaxed with tofacitinib (tof), ruxolitinib (rux), or vehicle (veh) and then treated with abacavir. (**C**) Ear thickness measurement. (**D**) Total number of MHCII^+^CD11b^+^CD11c^hi^ myeloid DCs per ear and Δ mean fluorescence intensity (MFI) of CD86 on ear DC by flow cytometry. (**E**) Total number of CD44^hi^CD62L^lo^CD8^+^ effector T cells in ear skin by flow cytometry. Pooled results are shown from 2 independent experiments; *n* ≥ 5 per group. (**C**) Two-way ANOVA with Bonferroni correction; (**D** and **E**) 1-tailed unpaired Welch’s *t* test; *P < 0.05; **P < 0.01; ***P < 0.001; ****P < 0.0001; #*P* < 0.05; ##*P* < 0.01; ###*P* < 0.001. (**F**) Patient clinical course schematic. (**G**) Representative clinical and histology images. Clinical exam showed diffuse maculopapular exanthem with subtle targetoid patches with central duskiness observed in areas of skin pain. Skin biopsy revealed spongiosis and vacuaolar interface dermatitis with perivascular lymphocytic infiltrate and eosinophils. (**H**) Representative IHC images (antigenic target, purple; counterstain, green). Scale bars: 100 μm.

## Version 1 04/08/2026

Electronic publication
