# Supplementary material for: JAK inhibition may prevent drug hypersensitivity reactions
Source: JCI Insight. 2026 Apr 8;11(7):e200914. doi: 10.1172/jci.insight.200914 (PMC13134734; doi:10.1172/jci.insight.200914)
Supplement: Supplemental data [file jciinsight-11-200914-s103.pdf]

Supplemental Material

Methods

Sex as a biological variable

Both sexes were included in human and mouse studies. Similar findings are reported for both sexes.

Mouse studies

HLA-B*57:01 C57Bl/6J transgenic (tg) mice were provided by D. Margulies (National Institute of Immunology, Allergy and Infectious Diseases, Bethesda, Maryland, USA) and M. Norcross (US Food and Drug Administration, Silver Spring, Maryland, USA). Heterozygous mice were bred inhouse by crossing with C57BL/6J mice (Jackson Laboratory). Male and female mice between 6 and 12 weeks of age were used in experimentation. Mice were phenotyped to confirm transgene expression (1).

All HLA-B*57:01^{pos} mice and HLA-B*57:01^{neg} littermate controls were treated by i.p. injection 5 days/week for 17 days with 3 mg of abacavir (ABC; Sigma Aldrich, #PHR1256) diluted in water for injection (1) and concurrently topically treated 3 days/week with 0.2 mg ABC in 30% ethanol on the left ear. All mice were depleted of CD4^{+} T cells by i.p. injection with 0.25 mg anti-CD4 mAb (Bio X Cell, clone GK1.5, #BE0003-1) in sterile PBS on days –3, +1, +4, and +7. For JAK inhibitor prophylaxis, subsets of mice were administered with tofacitinib (20 mg/kg; MedChemExpress, #HY-40354), ruxolitinib (30 mg/kg; MedChemExpress, #HY-50856), or vehicle (4% DMSO in 0.5% methylcellulose/saline; Sigma-Aldrich, #M0430) by oral gavage daily starting five days before abacavir treatment.

Mice were monitored for clinical signs of dermatitis and ear thickness measured by digital caliper (Mitutoyo Corp., Tokyo, Japan) every other day for the entirety of each study. Mice were harvested on day 22 at peak of disease (1, 2). A portion of ear was fixed in 10% neutral buffered formalin, embedded in paraffin, and sectioned at 4 microns for H&E staining by standard method. Remaining ear skin was cut into small pieces, then incubated in 3 mL HBSS (Thermo Fisher Scientific, #14175095) with 1 mg/mL collagenase A (Roche, #10103586001) and 40 µg/mL DNase I (Roche, #11284932001) at 37°C for 2 hours in a shaking incubator. To neutralize the collagenase, RPMI plus 10% FBS was added to the suspension and cells spun down. Cell pellets were resuspended in complete RPMI media then disaggregated through a 70 µm filter. Single-cell suspensions of ear skin were stained for dead cells with Zombie Violet™ Fixable Viability Kit (Biolegend, #423114) according to manufacturer's instructions. Cells were then treated with blocking mouse CD16/CD32 (Clone 2.4G2; BD Pharmingen, #553142), then were surface stained with combinations of the following: BV510-CD62L (clone MEL-14, Biolegend, #104441), BV605-CD86 (clone GL-1, 1:100, Biolegend, #105037), BV650-CD11b (clone M1/70, Biolegend, #101259), BV750-CD11c (clone N418, Biolegend, #117357), FITC-CD44 (clone IM7, Biolegend, #103022), PE-CD69 (clone H1.2F3, Biolegend, #104508), PE-Dazzle 594-CD8 (clone 53-6.7, Biolegend, #100762), PE/Cy7-CD3 (clone 17A2 , Biolegend, #100220), APC-CD40 (clone 3/23, Biolegend, #124612), AF700-MHCII (clone M5/114.15.2 , Biolegend, #107622), and APC/Cy7-CD45 (clone I3/2.3, Biolegend, #147717).

Human studies

Formalin-fixed paraffin embedded (FFPE) skin samples of MPE, DRESS and SJS/TEN were obtained from three sources. First, clinically obtained specimens were identified from 1/1/2000 – 12/31/2016 via pathology database searches at Boston Children's Hospital (BCH), Brigham and Women's Hospital (BWH) and Massachusetts General Hospital. All cases were clinically diagnosed by a board-certified dermatologist, had skin biopsy read as consistent with those diagnoses by a boarded dermatopathologist, and were vetted by a dermatologist with expertise in DHR (SJD). Secondly, skin samples were prospectively obtained from patients with MPE and SJS/TEN from BWH. Thirdly, clinically obtained skin biopsies from the two reported patients were used. Human skin discarded during plastic or dermatologic surgeries served as healthy controls.

Bulk Transcriptional Profiling

JAK/STAT transcriptional data in supplemental figure 2A are plotted from a previously described transcriptional profiling dataset of DHR and healthy control skin samples using a 200 NanoString Technologies custom designed gene panel (2).

Histology and Immunohistochemistry

Hematoxylin and eosin staining was carried out by standard technique. For immunohistochemistry (IHC), 5 µm FFPE skin sections were baked, deparaffinized and rehydrated. Sections underwent heat-mediated antigen retrieval with Tris-EDTA pH 9.0 or Citrate buffer pH 6.0, followed by treatment with BLOXALL® Endogenous Blocking Solution (SP-6000-100, Vector Laboratories), Avidin/Biotin blocking (SP-2001, Vector Laboratories), then blocking of non-specific protein binding using 5% Normal Donkey Serum (566460, Sigma-Aldrich). Sections were stained with primary

antibodies: phospo-JAK1 (PA5-104554, Thermo Fisher Scientific; Tyr1034, Tyr1035), phospho-JAK2 (PA5-114594, Thermo Fisher Scientific; Tyr1008), phospho-JAK3 (PA5-105892, Thermo Fisher Scientific; Tyr981), phospho-Stat1 (9167S, Cell Signaling Technology; Tyr701) and phospho-Stat2 (PA5-104922, Invitrogen; Tyr690). Sections were washed and stained with biotinylated anti-rabbit IgG (BA-1000, Vector Laboratories), then Streptavidin-HRP (SA-5004, Vector Laboratories). Slides were developed using ImmPACT® VIP Substrate Kit (SK-4605, Vector Laboratories) and counterstained with methyl green (H-3402-500, Vector Laboratories). Sections were imaged using the Mantra™ Quantitative Pathology Workstation and analyzed using InFORM analysis software (Akoya Biosciences). No primary and healthy control tissue served as negative controls. GVHD, psoriasis, and/or squamous cell carcinoma served as positive controls.
Activated protein expression was analyzed qualitatively and quantitatively. For quantification, IHC was scored blindly and independently by two observers using an arbitrary scale measuring protein expression from 0-3, with 0 meaning no expression, 1 mild, 2 moderate and 3 high. For each tissue section, the keratinocytes/epidermis and dermal inflammatory infiltrate were separately scored in each type of DHR, then averaged for an overall skin score.

Statistics
Mouse studies: Statistical analysis was performed using Graphpad Prism (v10). Significant differences between experimental groups were determined by two-way ANOVA with Bonferroni correction (group × time interaction; all pairwise comparisons were assessed, and only two relevant are displayed in the graph) or one-tailed unpaired Welch's t-test (only two pre-specified comparisons as displayed in graphs were tested) as indicated in figure legends. ns, not significant; $^{*}p < 0.05$; $^{**}p < 0.01$; $^{***}p < 0.001$; $^{****}p < 0.0001$; #P < 0.05; ##P < 0.01; ###P < 0.001. *Human studies*. Differential gene expression was performed with the DESeq2-v.1.26.0 R package as previously described (2). To correct for multiple comparisons, adjusted p-values (p_{adj}) were estimated using the false discovery rate. Log2FC > ± 1, $p_{adj} < 0.05$ was considered significant. Protein expression was assessed using a Mann-Whitney test when comparing two groups. When comparing more than two groups, a Kruskal-Wallis test was performed, and if significant, was followed by Dunn's test for multiple comparisons. P < 0.05 was considered statistically significant. Data were graphed using Graphpad Prism (v9).

Study Approval
The human research was approved by the institutional review boards at each participating site (Mass General Brigham IRBs 2016P001357, 2017P002826, 2018P001497, and BCH IRB-P00024905). Prospective study patients provided written informed consent before enrollment. Mouse experiments were performed in accordance with the guidelines put forth by the Center for Animal Resources and Comparative Medicine at Harvard Medical School (IACUC approval 2016N000070).

Data Availability
Values for all data points in graphs are reported in the Supporting Data Values file. Additional data are available from the corresponding author upon reasonable request. Only deidentified human data will be shared.

Acknowledgements
The authors acknowledge the following core facilities: The Specialized Histopathology Core, Dana-Farber/Harvard Cancer Center, supported in part by an NCI Cancer Center Support Grant 5P30CA06516, the Harvard Chan Bioinformatics Core with support from Harvard Catalyst, the Harvard Clinical and Translational Science Center (National Center for Advancing Translational Sciences, National Institutes of Health Award UL1TR002541) and financial contributions from Harvard University and its affiliated academic healthcare centers. The content is solely the responsibility of the authors and does not necessarily represent the official views of Harvard Catalyst, Harvard University and its affiliated academic healthcare centers, or the National Institutes of Health. We thank the Human Skin Disease Resource Center of Brigham and Women's Hospital and Harvard Medical School which provided healthy human control skin. The Human Skin Disease Resource Center is supported in part by NIAMS Resource-based Center Grant P30AR069625.

References
1. Cardone M, *et al.* A transgenic mouse model for HLA-B*57:01-linked abacavir drug tolerance and reactivity. *J Clin Invest.* 2018; 128(7):2819–2832
2. Shah PN, *et al*. Systemic and skin-limited delayed-type drug hypersensitivity reactions associate with distinct resident and recruited T cell subsets. *J Clin Invest*. 2024; 134:e178253

Supplemental Figure 1. JAK inhibitors prevent MPE-like dermatitis in mice. **(A)** Schematic of the prophylaxis protocol with JAK inhibitors (tofacitinib, ruxolitinib or vehicle control) in abacavir-induced MPE mouse model. Prophylaxis began 5 days before initiation of abacavir treatment. **(B)** Total number of $CD45^+$ leukocytes, $CD3^+$ T cells, $MHCII^+$ antigen presenting cells (APCs), and $MHCII^+CD11b^+CD11c^-$ monocytes/macrophages (mono/Mϕ) in ear, quantified by flow cytometry. Pooled results shown from two independent experiments, n ≥ 5 per group. P-values are determined by one-tailed unpaired Welch's t-test; **, p<0.01; ***, p<0.001.

Supplemental Figure 2. JAK-STAT pathways are activated in DHR skin. **(A)** JAK-STAT gene transcription in DHR compared to healthy control skin. Significance defined as Log2FC > ±1, $p_{adj}<0.05$. SJS/TEN n=13, DRESS n=6, MPE n=6, Healthy n=10. **(B, C)** Activated JAK-STAT protein expression by IHC staining (antigenic target, purple; counterstain, green) DHR n≥13 and Healthy n≥13. **(B)** Representative images shown. Black bar=100mm. **(C)** Quantification of IHC staining of all DHR compared to Healthy. Dotted lines show median. Two-tailed Mann-Whitney test; ***, p<0.001; ****, p<0.0001. **(D-F)** Quantification of IHC staining of individual types of DHR each compared to Healthy. IHC staining in keratinocytes, inflammatory infiltrate, and combined (expression quantified in keratinocytes and inflammatory infiltrate separately than averaged). SJS/TEN n≥5, DRESS n=4, MPE n=4, Healthy n≥13. Dotted lines show median; Kruskal-Wallis test comparing each form of DHR to healthy control, followed by Dunn's test for multiple comparisons. *, p<0.05; **, p<0.01; ***, p<0.001; ****, p<0.0001; ns, not significant.

Supplemental Figure 3. **Clinical Case 2.** A 69-year-old woman with history of vulvovaginal squamous cell carcinoma status-post surgical resection, cisplatin chemotherapy and radiation, then chronic myelomonocytic leukemia status-post hematopoietic stem cell transplant, received paclitaxel for radiation-associated angiosarcoma. **(A)** Schematic of clinical course. The patient developed rash and malaise after the 10th paclitaxel dose that improved slightly with prednisone 20mg daily for 3 days, but then worsened significantly after the 11th dose of paclitaxel. Exam revealed a maculopapular exanthem over the trunk and four extremities, with facial erythema and edema. There was no palm or sole involvement or follicular prominence. The patient endorsed oropharyngeal irritation and dysuria and had small superficial erosions on the hard palate and the vulva. There was no fever or lymphadenopathy. Two skin biopsies revealed vacuolar interface dermatitis with perivascular lymphocytic infiltrate and eosinophils. Laboratory analysis showed no eosinophilia or atypical lymphocytes. The patient had previously down-trending liver function tests, elevated from choledocholithiasis two weeks prior, that increased with her rash. The patient was diagnosed with possible DHR thought secondary to paclitaxel based on timing of the reaction. The differential diagnosis included GVHD though was less favored due to the temporal association with paclitaxel, the lack of palm/sole involvement, follicular prominence, gut involvement, or bilirubinemia, and the presence of eosinophils on skin biopsies. The patient improved with prednisone 0.5mg/kg/day which was slowly tapered. The patient was started on ruxolitinib 10mg orally twice daily prior to rechallenge with full-dose paclitaxel. The patient tolerated paclitaxel rechallenge without signs or symptoms of DHR. The patient ultimately self-discontinued ruxolitinib, tolerating 4 chemotherapy treatments thereafter without reaction, then underwent radical surgical excision with clinical and radiologic clearance beyond 36 months follow-up. **(B)** Clinical image. **(C)** Representative histology. **(D)** Representative IHC images (antigenic target, purple; counterstain, green). Solid black scale bar=100 mm. Dotted black scale bar=25 mm.

Supplemental Figure 4. Clinical Case 3. A 54-year-old female with metastatic uterine carcinosarcoma developed SJS/TEN to a platin agent and was successfully rechallenged with JAKi prophylaxis. **(A)** Schematic of clinical course. The patient developed a maculopapular rash two weeks after initiation of carboplatin, paclitaxel, and pembrolizumab, which

resolved with topical steroids. Following a second cycle of the same regimen, the rash recurred but responded to prednisone. Pembrolizumab was discontinued and replaced with trastuzumab. However, shortly after the third cycle, symptoms progressed to SJS/TEN. Consistent with clinical diagnosis, skin biopsy showed confluent, near–full-thickness keratinocyte necrosis with early subepidermal separation, "ghosted"/eosinophilic keratinocytes, and a pauci-inflammatory superficial perivascular infiltrate. Disease was initially presumed secondary to the pembrolizumab. Her SJS/TEN was treated with etanercept. The patient failed alternative chemotherapy so was rechallenged to carboplatin. One day later, she developed a rash concerning for early SJS clinically with histology showing a vacuolar/interface dermatitis pattern with basal vacuolar change with lymphocytes "tagging" the dermo-epidermal junction, scattered eosinophils within a superficial perivascular lymphocytic infiltrate, and pigment incontinence with melanophages. This reaction was aborted with systemic steroids. Her cancer continued to progress. Oncology deemed her only chance at survival was a platin agent. The patient initiated tofacitinib 5 mg twice daily, then tolerated two doses of cisplatin. Tofacitinib was then held due to upper respiratory infection. Two days later, the patient developed a DHR with histology showing mild spongiotic pattern associated with drug hypersensitivity: minimal intercellular epidermal edema, sparse superficial perivascular lymphocytic infiltrate with rare eosinophils. No keratinocyte necrosis. This reaction was managed with topical steroids. Kidney disease (unrelated to the DHR) prevented further chemotherapy and she was transitioned to hospice care. **(B)** Representative clinical images. **(C)** Representative histology. Solid black scale bar=50 mm.

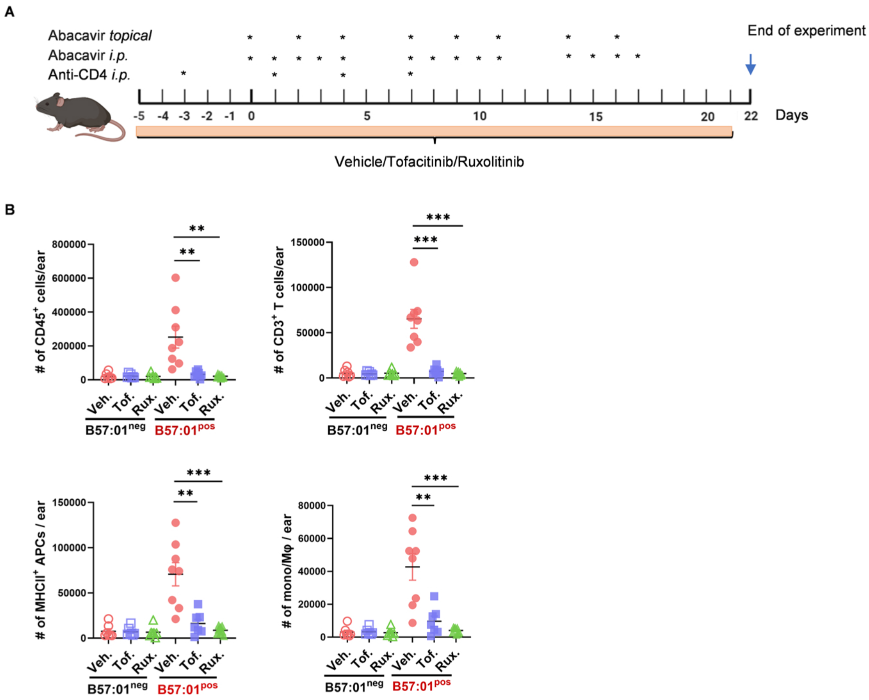

Supplemental Figure 1

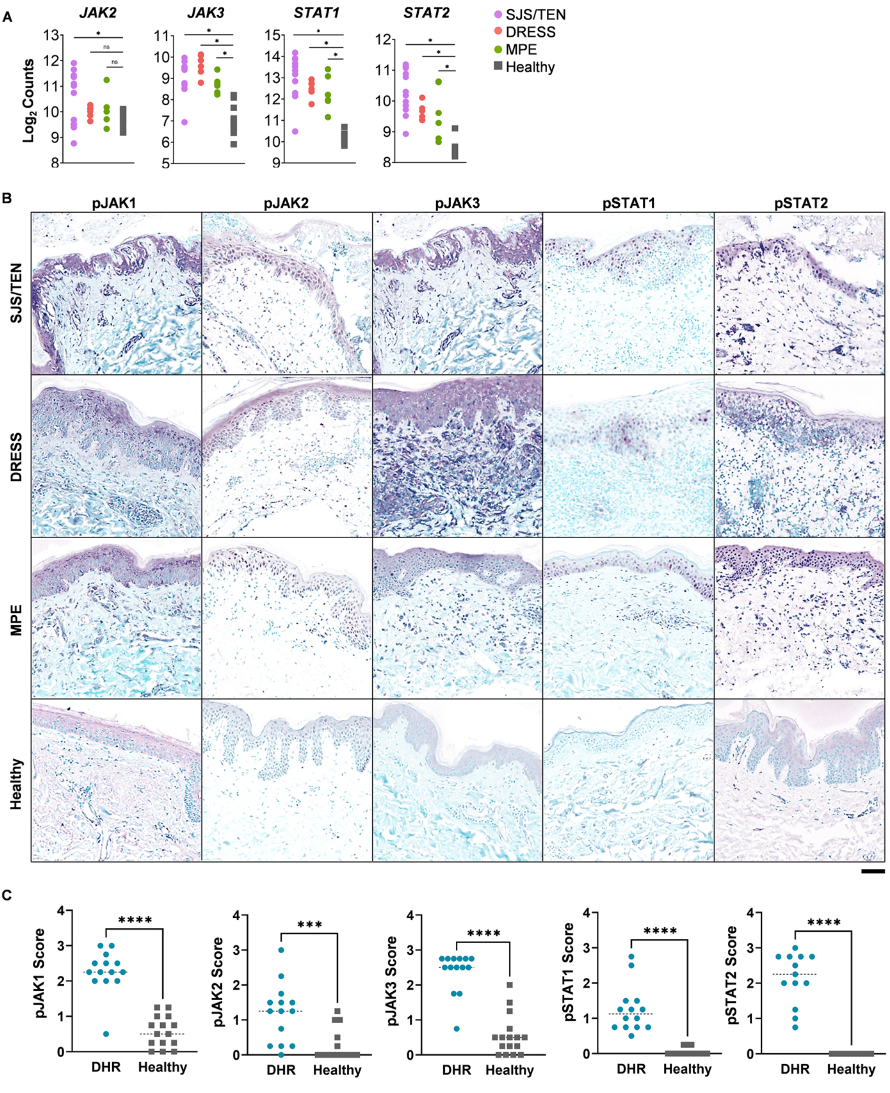

Supplemental Figure 2

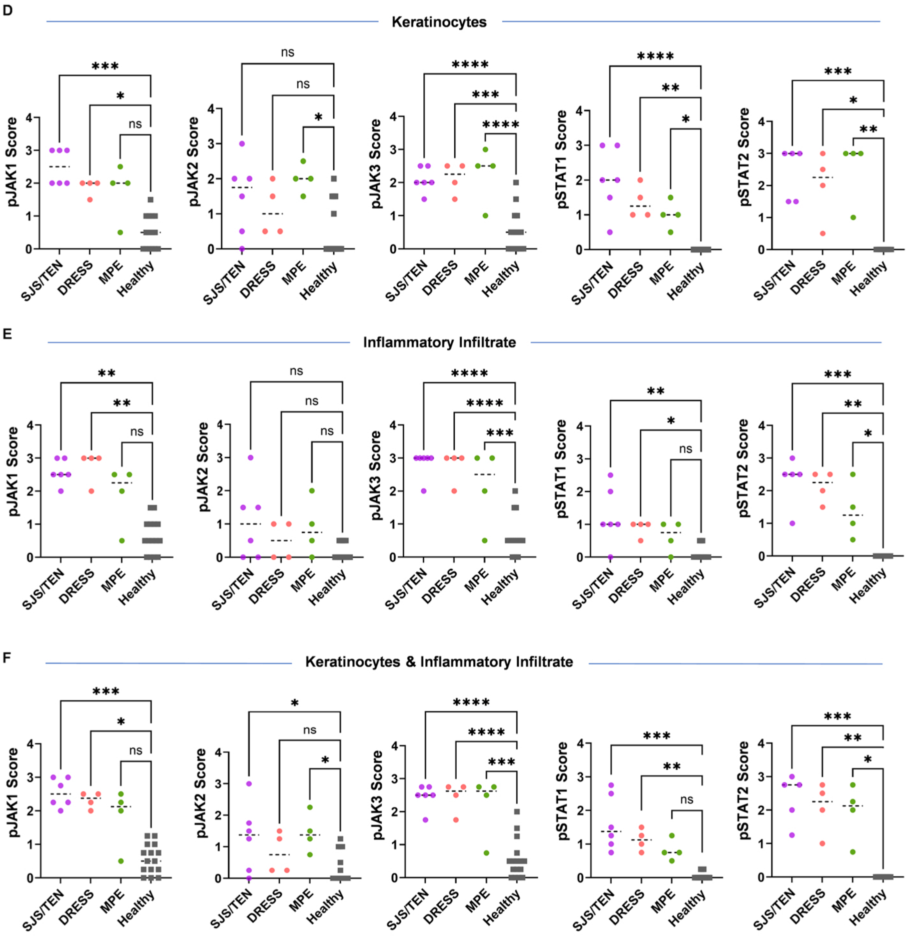
D
Keratinocytes
pJAK1 Score
pJAK2 Score
pJAK3 Score
pSTAT1 Score
pSTAT2 Score
SJS/TEN
DRESS
MPE
Healthy
E
Inflammatory Infiltrate
F
Keratinocytes & Inflammatory Infiltrate
ns

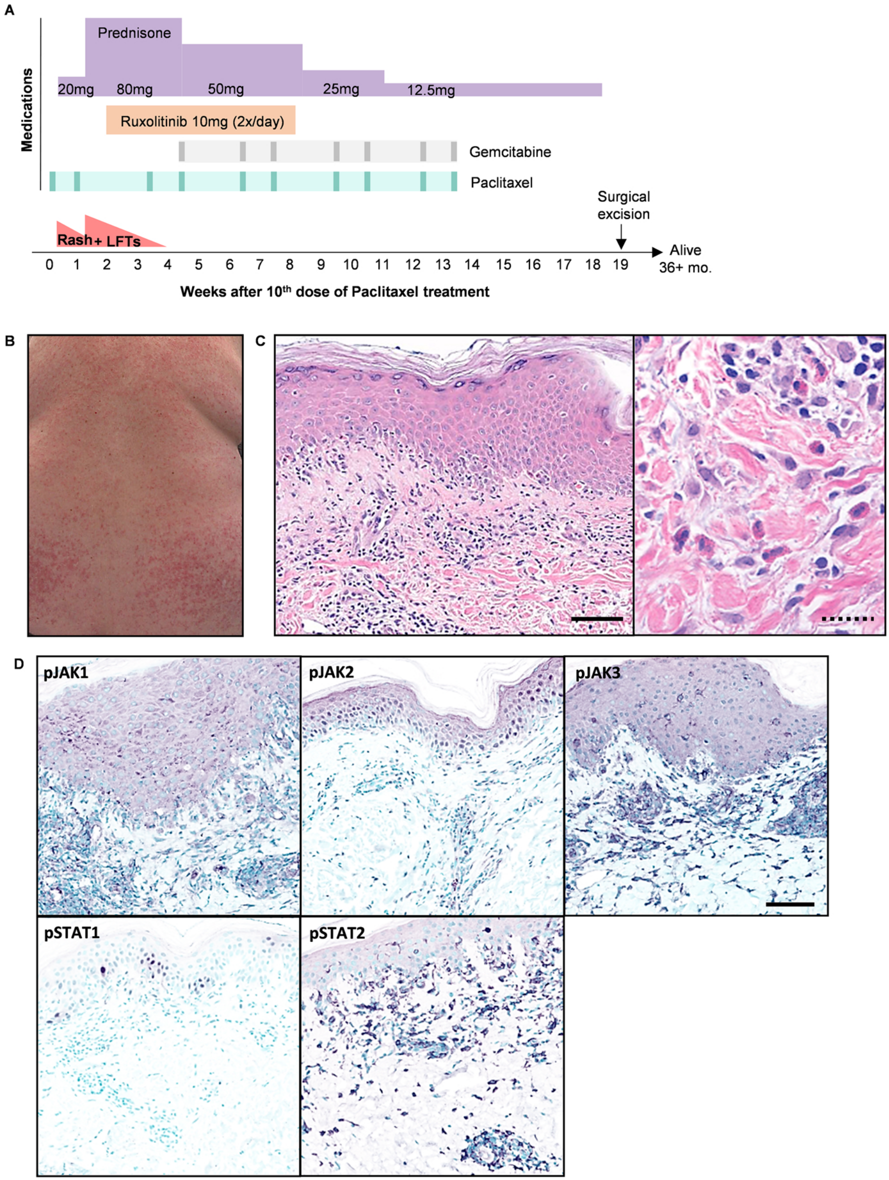

Supplemental Figure 3

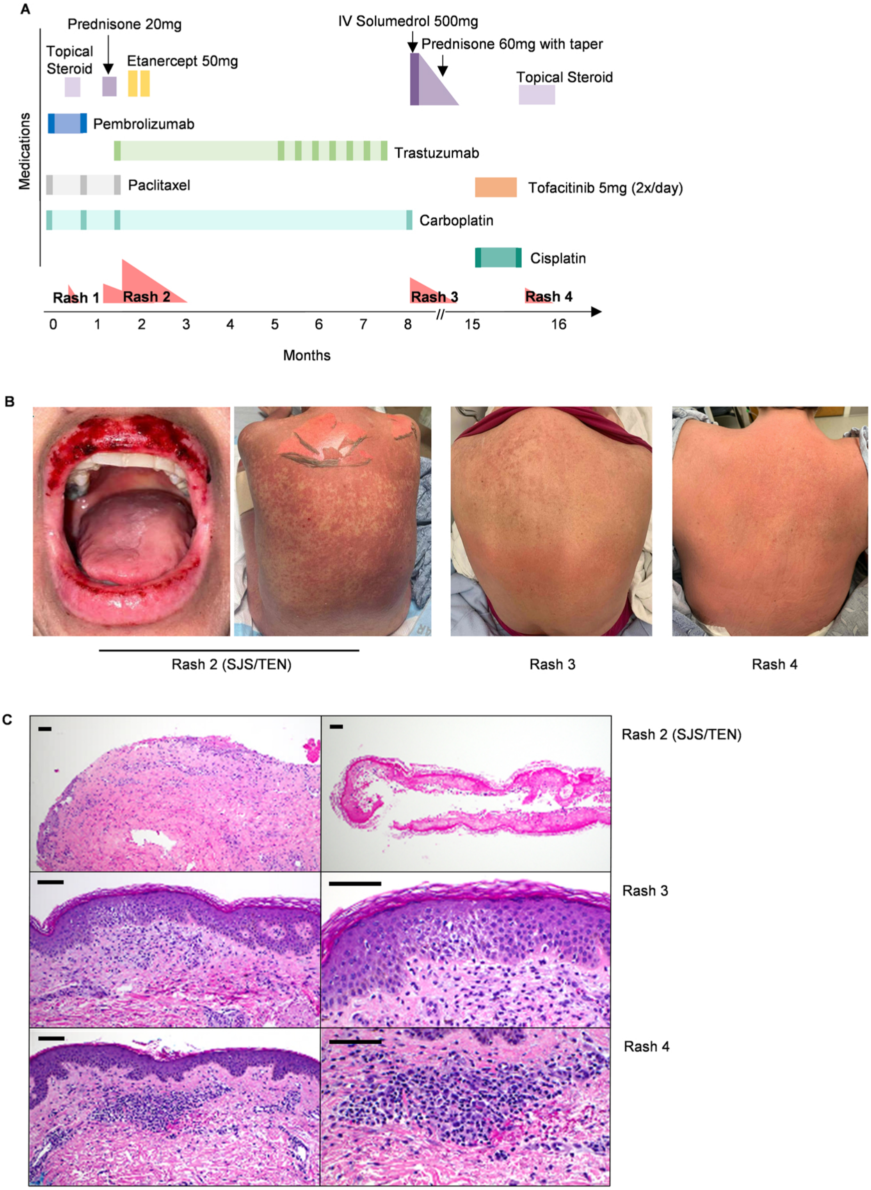

Supplemental Figure 4
